# High-Protein or Low Glycemic Index Diet—Which Energy-Restricted Diet Is Better to Start a Weight Loss Program?

**DOI:** 10.3390/nu13041086

**Published:** 2021-03-26

**Authors:** Ewa Waliłko, Małgorzata Napierała, Marta Bryśkiewicz, Aneta Fronczyk, Liliana Majkowska

**Affiliations:** Department of Diabetology and Internal Diseases, Pomeranian Medical University, 72-010 Szczecin-Police, Poland; ewalilko@o2.pl (E.W.); mal.napierala@gmail.com (M.N.); martab@pum.edu.pl (M.B.); anetafk@wp.pl (A.F.)

**Keywords:** energy-restricted diet, jump-start diet, high-protein diet, low-glycaemic index diet, crossover trial

## Abstract

Background: To date, no crossover studies have compared the effects of high-protein (HP) and low glycemic index (LGI) diets applied as starting energy-restricted diets. Methods: Thirty-five overweight or obese volunteers with sedentary lifestyles aged 41.4 ± 11.0 years, with body mass index (BMI) of 33.6 ± 4.2 kg/m^2^, without diabetes, completed an 8-week randomized crossover study of an energy-restricted diet (reduction of 30%; approximately 600 kcal/day). The anthropometric parameters, body composition, 24 h blood pressure, and basic metabolic profile were measured at baseline and after completing the two 4-week diets; i.e., the HP (protein at 30% of the daily energy intake) or LGI diet, followed by the opposite diet. All subjects maintained food diaries and attended six counselling sessions with a clinical dietitian. Results: The final weight loss was not significantly different when the HP diet was used first but was associated with a greater loss of fat mass: 4.6 kg (5.8; 3.0 kg) vs. 2.2 (4.5; 0.8); *p* < 0.025, preserved muscle mass, and reduced LDL-cholesterol. Conclusions: A short-term HP diet applied as a jump-start diet appeared to be more beneficial than an LGI diet, as indicated by the greater fat mass loss, preservation of muscle mass, and better effects on the lipid profile.

## 1. Introduction

Dietary interventions for weight loss should achieve a state of negative energy balance [1,2]. The goals set for an overweight or obese person should be rational and achievable. The food portions for adults should be reduced by 15–30% of caloric intake from the habitual diet, which corresponds to a deficit of 600–700 kcal/day. As a result, a body weight loss of approximately 0.5 kg/week can be expected [3]. In addition to providing adequate energy, special attention should be given to the types of products included in the diet. A poorly balanced diet can lead to deficiencies of many nutrients, including both macronutrients and micronutrients, or dysregulation of the lipid and glucose metabolism. Carbohydrates should constitute 55–60% of the total daily energy intake, proteins 12–15%, and fats should not exceed 25–30% [4]. According to certain European guidelines, the dietary references for carbohydrates are 45–65% and are 10–20% for protein [5,6]. Many popular energy-restricted diets are based on manipulation of the macronutrient contents or restrictions of specific foods. The prescription for hypocaloric intake associated with such changes might be more effective for weight loss.

Two types of diet deserve special attention: a high-protein diet (HP) and a low glycemic index diet (LGI). Both diets, easy to follow, are becoming increasingly popular for weight loss. Low-carbohydrate diets with an increased fat content (HF diets) should not be recommended due to their adverse effects on metabolism [7,8].

An HP diet is a variant of a low-carbohydrate diet and is characterized by an increased intake of foods rich in protein. In a balanced “healthy” diet recommended for most healthy subjects, approximately 15% of energy comes from protein, while in high-protein diets, this rate is ≥16%, and sometimes higher than 30% [9,10,11,12]. In energy-restricted HP diets for body weight loss, 25–30% of the energy is supplied by protein. These diets are relatively high in protein; however, in absolute terms they contain usually 0.8–1.2 g/kg of body weight/day, similar to the normal-protein diet in neutral energy balance [11,13].

One of the mechanisms proposed to be responsible for the weight-loss-promoting effect of HP diets is an increase in diet-induced thermogenesis [14]. Additionally, during weight loss, HP diets preserve lean body tissue, the major determinant of resting and diurnal energy expenditure [11]. The satiety and fullness effect of protein ingestion appears to play a role in a decrease of energy intake [15]. Some reports indicate that an HP diet is associated not only with significant weight loss and improvement of body composition but also with positive effects on the lipid metabolism, and may reduce the risk of cardiovascular diseases and lower blood pressure [10,11,12,16].

While the short-term effect of HP diets in which ≥ 20% of the energy is derived from protein may offer advantages regarding weight loss and body composition, the long term effects of energy-restricted diets with different ratios of macronutrients may promote similar effects [17,18,19]. Due to these observations, an HP diet appears to be a better tool to jump-start weight loss rather than a long-term diet.

An LGI diet does not differ from a normal balanced diet in terms of the total carbohydrate, protein, and fat content. The main goal of an LGI diet is to eliminate food products containing carbohydrates that cause a rapid increase in the blood glucose and insulin concentrations. Products containing carbohydrates should have a low glycemic index (GI), not higher than 55. Meals with a high-glycemic index/load of carbohydrates stimulate significant postprandial insulin secretion [20].

Acute glucose-stimulated insulin hypersecretion was correlated with weight gain [21]. In mixed diets with a substantial amount of carbohydrates and fat, like current Western diets, insulin hypersecretion promotes fat storage and plays a key role in obesogenesis [22,23]. Foods containing carbohydrates with a low GI are more satiating, reduce the number of snacks, and curb appetite so that the subsequent meal is less caloric [24,25].

In subjects on LGI diets, reductions in body mass, body mass index (BMI), and waist circumference as well as improved lipid metabolism, reduced insulin resistance, normalization of glucose levels, and reduced risk of diabetes in later life have been observed [26,27,28]. A recent meta-analysis demonstrated that LGI diets were beneficial for weight loss in people with normal glucose tolerance, while in subjects with type 2 diabetes or those with impaired glucose tolerance, weight loss was not observed [28].

Regardless of the type of diet, personalized modification and adherence to the diet are of great importance [29,30]. A higher level of dietary adherence, rather than the type of diet, may be the key factor in predicting the success of weight loss in overweight and obese subjects [31].

Intervention studies conducted to date investigating the two aforementioned energy-restricted diets compared their effectiveness to that of other diets used in parallel control groups. However, no previous studies compared the effects of HP and LGI diets in the same group of overweight or obese subjects beginning a weight loss program controlled by a dietitian.

The aim of this randomized crossover study was to assess the short-term effects of two energy-restricted diets, an HP diet and an LGI diet, on body mass and body composition, blood pressure, and metabolic parameters in the same group of overweight or obese subjects who started their weight loss program with a highly personalized diet under the supervision of a clinical dietitian. 

## 2. Materials and Methods

### 2.1. Subjects

The study group comprised overweight or obese subjects with sedentary lifestyles, interested in reducing their body weight. Diabetes mellitus, impaired renal function (estimated glomerular filtration rate e-GFR < 60 mL/min), electrolyte disorders, severe systemic diseases, such as unstable coronary disease, heart failure, arrhythmias, uncontrolled hypertension, secondary hypertension, a history of stroke, liver diseases, neoplastic diseases, chronic hematological diseases, uncontrolled hypothyroidism or hyperthyroidism, gout, mental disorders, pregnancy, and lactation were considered as exclusion criteria. Subjects who required a modification of the treatment of chronic diseases or did not adhere to the recommendations specified in the study protocol were excluded from the trial. Participants were enrolled after being provided full information on the purpose and the protocol of the study, and all subjects provided written informed consent to participate in the study. The study was approved by the Bioethics Committee of the Pomeranian Medical University in Szczecin (KB-0012/01/11).

Fifty subjects were enrolled in the study (43 women and 7 men). From this group, 15 (12 women and 3 men) dropped out or were excluded from the trial due to non-adherence (non-attendance at the planned visit, omitting any counselling session with dietitian, as well those who did not reach the calorie intake recommended by the protocol due to excessive consumption of sweetened beverages or alcohol). Thirty-five subjects (31 women and 4 men) with a mean age 41.5 ± 11.0 years and a mean BMI of 33.6 ± 4.2 kg/m^2^ completed the trial.

### 2.2. Study Protocol

The study subjects participated in an 8-week intervention, which consisted of two different 4-week energy-restricted diets with reduced calorie intake (approx. 600 kcal per day) and unchanged physical activity. The subjects were randomized into the crossover trial in which they consumed a hypocaloric HP or LGI diet for a 4-week period followed by 4 weeks on the opposite diet. The diets differed in the protein content and GI. In the HP diet, the target was for 30% of the energy intake to be derived from protein, 40% from carbohydrates, and 30% from fat with no specific instructions regarding the GI.

In the LGI diet, the protein intake was planned to account for 20% of the energy intake but no less than 0.83 g/kg of body mass [6], carbohydrates for 50%, and fat for 30%, and the subjects were instructed and advised to consume LGI foods. Foods with a GI < 55 were considered low GI foods [32]. For both diets, a high intake of vegetables (mainly raw) and salads was encouraged, and the intake of added sugars and alcohol was discouraged. The HP-LGI group consisted of 17 subjects (15 women and 2 men) who followed the HP diet first, and then were switched to the LGI diet. In the LGI-HP group, consisting of 18 participants (16 women and 2 men), the LGI diet was given first, followed by the HP diet.

A wash-out period between the diets was not used to prevent interruption of the weight loss process. All participants attended individualized dietary education sessions with a clinical dietitian. They were required to attend six counselling sessions, including two visits for each period, during which food diaries were given/assessed and intensive dietary guidance was provided. The subjects maintained a diary of their food intake at baseline according to their habitual diet and for each 4-week energy-restricted diet period.

For each period, the participants recorded all beverages and foods consumed on 3 different days (2 weekdays and a weekend day). The amounts of consumed foods were recorded in household units, by volume or weighed with a scale. A trained dietician assessed the food diaries and gave detailed instructions for personalized diets with a reduced calorie intake compared with the subjects’ habitual diet and high/normal protein content or GI.

**Figure 1:**
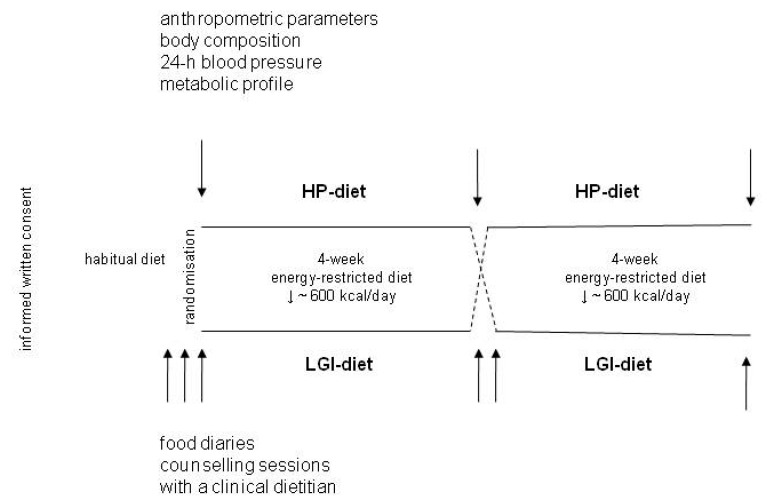
The crossover design of 8-week intervention with energy-restricted high-protein (HP) and low glycemic index (LGI) diets.

The data from the diaries were analyzed using the DIETETYK v.2.0 software (Food and Nutrition Institute, Warsaw, Poland) and referenced to the current standards recommended by the Food and Nutrition Institute in Warsaw [33]. Based on the diary records of each participant, the daily intake of energy and macronutrients was assessed before the intervention and after each 4-week period when one of the diets was applied. The design of the diet program is shown in Figure 1.

### 2.3. Assessment of the Effects of Dietary Intervention

During the trial, each participant was tested three times for anthropometric parameters, body composition, 24 h blood pressure, and a basic laboratory panel before starting the energy-restricted diet and after each 4-week period on one of the two diets (Figure 1). The anthropometric parameters, i.e., the body weight, height, body mass index (BMI), waist and hip circumference, and waist/hip ratio (WHR), were measured using calibrated scales and anthropometric tape. The body composition was analyzed using the bioimpedance technique with a Tanita analyzer model BC418 (Tanita Corp., Tokyo, Japan) according to the manufacturer’s recommendations. It was measured in patients in the fasting state after emptying their bladder and after a 15 min rest.

The assessed parameters included the total body fat mass (FM), fat-free mass (FFM), muscle mass (MM), and total body water (TBW). The blood pressure was measured for 24 h using a SpaceLabs monitor model 90,207 (SpaceLabs Inc., Redmond, WA, USA), and the diurnal systolic blood pressure (SBP), diastolic blood pressure (DBP), and mean arterial pressure (MAP) were recorded. Laboratory tests were performed on fasting venous blood samples taken in the morning. Standard biochemical analyses for glucose, lipids (the total cholesterol, LDL: low-density lipoprotein cholesterol, HDL: high-density lipoprotein cholesterol, and triglycerides), alanine and aspartate aminotransferases (ALAT and ASPAT), uric acid, and creatinine, were carried out at the University Hospital Laboratory, MEDIS.

### 2.4. Anthropometric Data, Blood Pressure, and Laboratory Parameters at Baseline

The baseline anthropometric parameters, body composition, blood pressure, and laboratory results of the investigated subjects are presented in Table 1.

### 2.5. Diet Control

The records from the food diaries maintained by the study participants were used to estimate the composition of their habitual diet before the intervention and during the use of both weight-reducing diets. Our analysis of the food diary data is shown in Table 2. Calorie intake was reduced by approximately 30% on both energy-restricted diets. The macronutrient content achieved during the HP diet was consistent with the plan. On the LGI diet, the carbohydrate intake was lower by 5%, and the protein intake was higher by 4% than planned, although still in the recommended range given in g/kg of body mass.

### 2.6. Statistical Analysis

The main hypothesis of this study stated that effects of high-protein (HP) and low glycemic index (LGI) diets applied as starting energy-restricted diets, measured as changes in patients’ body mass and fat mass, are different. The sample size sufficient to provide 80% statistical power for the detection of real differences between the HP-LGI and LGI-HP groups equal to one standard deviation of a parameter at the 0.05 significance level was calculated to be 17 subjects in each group. The values of the quantitative variables are presented as the arithmetic mean ± standard deviation (SD) or as the median (lower quartile and upper quartile) for variables with an SD exceeding the mean value.

Since the distributions of most of the quantitative variables were significantly different from the normal distribution (Shapiro-Wilk test), non-parametric tests were used: the Mann-Whitney U test for comparisons between independent groups and the Wilcoxon signed-rank test for comparisons of paired measurements. *p* < 0.05 was considered statistically significant. The statistical analysis was performed using STATISTICA v.10 (StatSoft Polska, Krakow, Poland).

## 3. Results

### 3.1. Body Mass and Body Composition on Energy-Restricted Diets

The changes in body mass and body composition during the 8 weeks of the energy-restricted diets are presented in Table 3.

**Figure 2:**
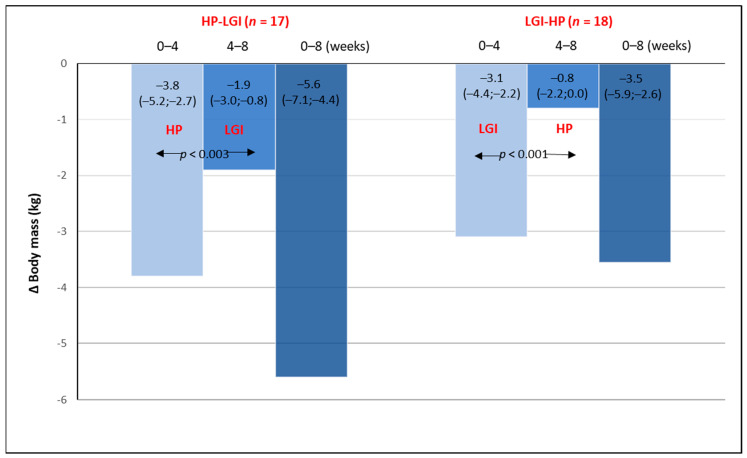
The changes in body mass (∆ body mass) during the 8-week period of energy-restricted diets: 4-week high protein (HP) or low glycemic index (LGI) diet given in a random order and followed by the opposite diet.

Regardless of the type of diet, the weight loss during the first 4 weeks of the intervention was comparable in both groups and significantly greater than that in the subsequent 4 weeks on the second diet (Figure 2). In the HP-LGI group, the weight loss after 8 weeks of the trial was slightly but not significantly greater (*p* = 0.07) compared with the LGI-HP group (Figure 2).

**Figure 3:**
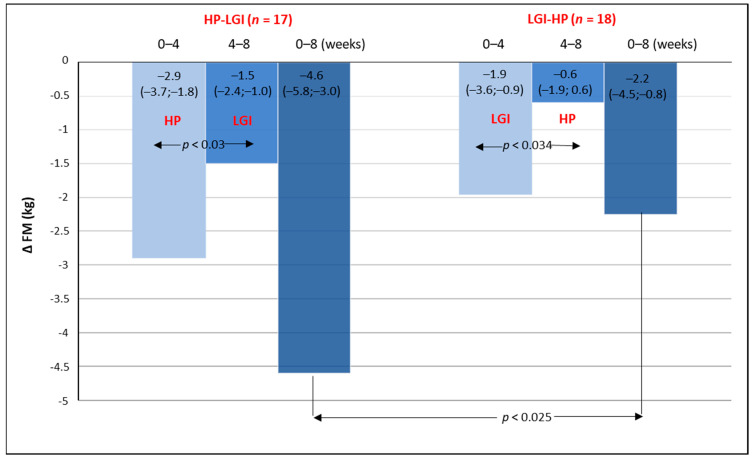
The changes in fat mass (∆FM) during the 8-week period of energy-restricted diets: 4-week high protein (HP) or low glycemic index (LGI) diets given in a random order and followed by the opposite diet.

A significant reduction in fat mass was observed in all participants. In both groups, the fat mass reduction was significantly greater during the first 4 weeks compared with the subsequent 4 weeks of the study (Figure 3). After 8 weeks of the intervention, the reduction of fat mass in the HP-LGI group was significantly greater compared with that in the LGI-HP group: 4.6 kg (5.8; 3.0 kg) vs. 2.2 (4.5; 0.8); *p* < 0.025.

In the HP-LGI group, the fat-free mass did not change significantly after the first 4 weeks of the HP diet (Table 3). After the subsequent 4 weeks of the LGI diet, no significant differences were found compared to the values obtained after the HP diet was completed. However, a significant reduction in fat-free mass compared to the baseline values was observed after 8 weeks of energy-restricted diets.

During the entire intervention period, no significant changes in muscle mass were observed in the HP-LGI group (Table 3). In the LGI-HP group, a significant reduction in the fat-free mass and muscle mass was observed after the first 4 weeks of the LGI diet. This difference persisted after 8 weeks of the study, both for the fat-free mass and muscle mass; however, the HP diet applied between weeks 4 and 8 had no significant effect on either of these parameters.

A significant increase in the total body water was observed in both groups (Table 3). In the HP-LGI group, an increase was observed both after 4 weeks and 8 weeks of the study. In subjects from the LGI-HP group, no significant changes were found after 4 weeks; however, the total body water after 8 weeks was significantly higher than it was at the baseline.

In both examined groups (HP-LGI and LGI-HP), a decrease in the waist and hip circumference was observed after the intake of both the first and second diets and after 8 weeks of their combination but had no effect on the WHR. The mean WHR before, during, and at the end of the trial in both groups was 0.8 ± 0.0.

### 3.2. Twenty-Four Hour Blood Pressure Monitoring on Energy-Restricted Diets

In the HP-LGI group, there were no significant changes in the SBP, DBP, or MAP measured for 24 h through 8 weeks of the trial. In the LGI-HP group the values of blood pressure measured at the baseline and after 4 weeks were similar; however, after the HP diet applied between weeks 4 and 8, the subjects’ blood pressure was significantly lower than it was after 4 weeks of the previous LGI diet: SBP 117 ± 10 mmHg vs. 122 ± 13 mmHg (*p* < 0.01), DBP 71 ± 9 mmHg vs. 75 ± 8 (*p* < 0.03), and MAP 88 ± 9 vs. 91 ± 9 mmHg (*p* < 0.02). These values did not differ significantly from the baseline values.

### 3.3. The Metabolic Profile on Energy-Restricted Diets

Changes of the metabolic profile in both groups are shown in Table 4. The fasting blood glucose levels and triglyceride levels did not change significantly during the study and did not differ between the study groups. The total cholesterol levels in both groups decreased significantly after the first 4 weeks of an energy-restricted diet and remained unchanged after the next 4 weeks of the opposite diet. The HDL cholesterol in both groups and LDL cholesterol in the LGI-HP group did not change significantly. The LDL cholesterol in the HP-LGI group decreased significantly after the first 4 weeks of the HP diet, from 3.3 ± 0.9 mmol/L at the baseline to 2.8 ± 0.8 mmol/L (*p* < 0.01) but increased to 3.0 ± 1.0 mmol/L after 4 weeks of the subsequent LGI diet (*p* < 0.04). Liver enzymes, including ALAT and ASPAT, measured after 4 weeks of each dietary intervention did not change in either of the groups. No changes were observed in the serum creatinine levels. The level of uric acid in the HP-LGI group was not changed; however, in the LGI-HP group, the level was reduced significantly compared to the baseline value. A reduction was recorded after the first 4 weeks and after the subsequent 4 weeks of the second diet.

## 4. Discussion

It is not clear whether consumed calories provided by different foods are equal or if some foods are more obesity promoting. Regarding dietary interventions for weight loss, it is also not clear whether negative energy balances attained on different diets are similarly effective or whether the manipulation of the macronutrient content in isocaloric diets may promote better results [2,17,19].

Studies comparing the effects of diets with increased protein content or a low glycemic index are usually conducted in parallel groups of subjects on ad libitum diets [11,28]. Intervention studies on the effects of energy-restricted HP and LGI diets with a crossover design are scarce. These trials last for several days to several weeks and compare the effects of diets with different protein intake and diets with low and high GIs [11,28]. To date, none of the crossover studies have compared the effects of a hypocaloric HP diet and a hypocaloric LGI diet with the standard protein content used as the starting intervention for weight loss in overweight or obese subjects.

The DioGenes study suggested similar positive effects from a high-protein diet combined with a high GI and a diet with a standard protein content and low GI in the first 6 weeks of the intervention [34]. It should be noted, however, that this intervention was introduced after a primary 8-week hypocaloric diet (800 kcal/day), which led to significant weight loss (approximately 11 kg on average). In addition, both diets were ad libitum diets used in different groups of subjects observed in parallel.

Both energy-restricted diets applied in our study as starting interventions for the first 4-week period were similarly effective in promoting the loss of body weight and fat mass. However, on the HP diet, the loss of 3.8 kg of body weight was more clearly related to the loss of fat mass (76%), while, in the LGI group, the loss of 3.1 kg of body mass was associated with a lower contribution of diminishing fat mass (61%).

In the group of subjects who applied the HP diet at the beginning of the dietary intervention, the loss of fat mass after 8 weeks was significantly greater than in the group starting with the LGI diet and was responsible for 82% of the total loss of weight (4.6 kg of 5.6 kg). The HP diet given first was more beneficial for a lower reduction in fat-free mass and preservation of muscle mass. These positive effects were maintained for the next 4 weeks of the LGI diet.

The preservation of muscle mass might be crucial for the maintenance of higher resting and 24-h energy expenditure and a greater energy deficit over time, which promotes a greater loss of body weight and fat mass [35,36]. The LGI diet prescribed as the first diet caused a significant loss of fat-free mass, muscle mass, and the persistence of these effects later with the HP diet. The observations of our study indicate that the HP diet given as a jump-start diet appeared to be more effective and beneficial compared with the LGI diet.

The energy-restricted HP diet applied in our study was high protein in relative terms, i.e., it constituted 30% of the energy supply; however, in absolute terms, it implied a normal protein intake, which was 1.0 g/kg of body weight. The same was observed in other energy-restricted HP diets, which are erroneously perceived as diets with doubled protein intake. Such diets are relatively high in protein (the percentage of energy from protein is 20–30%), but since they are energy-restricted, they usually contain a normal absolute amount of protein 0.8–1.2 g/kg of body weight [11,13,37].

Normal protein intake is required for body weight loss and weight maintenance. However an energy-restricted “normal-protein” diet providing at least 0.8 g/kg of body weight is sufficient for substantial weight loss. The protein intake of 1.2 g/kg is more beneficial for the body composition (the effect of fat-free mass), improved metabolic profile, sustained resting energy expenditure, and weight maintenance [13,37]. These facts may explain the better results observed in our study for the subgroup in which the HP diet was applied first.

The protein intake in this group was lower than indicated above but still higher than in the LGI group with protein intake of approximately 0.8 g/kg. Even such a small difference in the absolute and still normal protein intake may be of great importance for the beneficial effects on body composition and lipid profile. In many of the analyzed energy-restricted, so-called, normal protein diets compared with reductive HP diets, the protein intake was below 0.7 g/kg of body weight and, in some of them, as low as 0.61–0.55 g/kg [11].

Our findings on the preservation of fat-free mass and muscle mass are slightly different from those observed in other studies comparing diets with high and standard protein contents. A systematic review and meta-analysis of studies that compared energy-restricted HP diets with standard-protein diets with a mean duration of 12 weeks revealed that an HP diet led to a greater reduction in body weight, fat mass, and triglycerides, and a lesser reduction in fat-free mass [11]. Studies with longer and shorter durations showed no significant differences for the majority of these outcomes except for the fat-free mass. A significantly lower reduction of fat-free mass was seen only in HP diets with a longer duration (≥ 12 weeks) and was not observed in studies that were shorter than 12 weeks. The mean dietary macronutrient contents of the HP diets in the analyzed studies were very similar to that in our study: 30.5% protein, 41.6% carbohydrates, and 27.8% fat for HP diets [11].

It is possible that the rapid effect related to the preservation of muscle mass observed in our study for the HP diet applied as the initial intervention may result from the fact that the diet was highly personalized for each subject and supervised by an experienced clinical dietitian; furthermore, all participants had regular and frequent educational visits regarding their diets.

The increased protein intake during the early phase of calorie restriction may be beneficial for muscle mass preservation and may elicit further weight loss and fat mass reduction. A positive outcome at the beginning of weight loss is extremely important for further patient motivation and adherence to a proper diet in the following weeks and months. The potential physiological mechanism of the beneficial effect of protein in the diet can be explained by a planned crossover trial investigating the short-term use of diets with high and standard protein contents, the protocol of which was recently published [38].

In our study, no changes in the fasting glucose or triglycerides were observed after each 4-week period in which one of the two diets was followed or after 8 weeks of the two diets combined. Very similar data on glycaemia during the HP diets were reported from a meta-analysis of previous studies [11]. Most of these studies (10 out of 12) were also conducted in subjects without diabetes. The duration of the HP diets and compared control diets had no effect on the glucose levels. In contrast to our study, the analyzed HP diets, including those followed for short periods of time, had more positive effects on triglyceride levels. However, we may assume that the duration of the starting diet in our study was too short to observe a similar effect. Because of the effects on post-prandial blood glucose and insulin excursions, low GI diets have been suggested to lower fasting glucose. However, a systematic review and meta-analysis of LGI diets used as interventions for obesity shows that the low GI diets were not associated with a lower fasting glucose [28]. The wholegrain diet characterized by low GI, applied for a quite long time (12 weeks) in individuals with metabolic syndrome, also did not affect plasma concentrations of glucose, insulin, and triglycerides measured at fast [39]. Interestingly, postprandial insulin and triglyceride responses were significantly lower at the end of the intervention in the wholegrain group compared to the parallel control group whereas there was no change in postprandial response of glucose.

This study is the first to compare short-term energy-restricted diets—HP and LGI diets, used as jump-start diets for a weight loss program, compared in a randomized crossover design. The caloric restriction in both diets was relatively low in comparison to the baseline diet of the participants; thus, these diets were easy to follow.

The proportions and intake of macronutrients were consistent with the nutritional recommendations for “healthy” diets. Both the HP and LGI diets were highly personalized for each participant interested in weight loss and were based on data obtained regarding their habitual diets. Both diets included products that were used in real life without any artificial supplements. All participants were carefully assisted by a highly trained clinical dietitian.

The number of participants of this study was relatively low, but quite typical for such kinds of studies. A systematic review and meta-analysis of LGI diets used as an intervention for obesity showed that the number of subjects in crossover studies with energy restriction was almost identical, or sometimes even lower, compared to the number of participants in our study [28].

Our randomized crossover study was not a blind study due to the fact that dietary manipulation of the protein composition and GI cannot be accomplished with a blinded design when real foods are used. Additionally, the diets of all participants were highly individualized and used at home, in a realistic setting. The investigator, a clinical dietitian, and the subjects were aware of the diet that had been assigned. Both start diets were rather short and the subjects changed to the opposite diet after 4 weeks. The design of the trial was planned in this way as it was clear that calorie-restricted diets with the prospect of eating very similar foods for long time cannot be maintained with high adherence. Food diaries from the period of both restricted diets included only 3 days of the intervention diets but seemed to confirm high adherence to the recommended intake of macronutrients.

Even if these records include slightly misreported data, adherence to a diet was not the main goal of the study. The interpretation of the results and practical implications would benefit from a comparison of the data obtained in the HP-LGI and LGI-HP groups to the results in subjects following only a personalized HP or LGI diet, each applied for 8 weeks. For organizational reasons, it was not possible to conduct this type of parallel study in our research center.

## 5. Conclusions

A short-term HP diet applied at the beginning of a weight loss program appeared to be more beneficial than an LGI diet, showing greater fat mass loss, preservation of muscle mass, and better effects on the lipid profile.

## Figures and Tables

**Table 1:** The baseline characteristics of the investigated subjects.

Variable	Total Group *n* = 35	HP-LGI Croup *n* = 17	LGI-HP Group *n* = 18	Statistics
**Anthropometric Parameters**				
Age (years)	41.5 ± 11.0	40.7 ± 12.7	42.1 ± 9.4	ns
Body mass (kg)	92.9 ± 15.3	94.1 ± 13.6	91.7 ± 17.1	ns
BMI (kg/m^2^)	33.6 ± 4.2	33.8 ± 4.0	33.4 ± 4.5	ns
WHR	0.80 ± 0.0	0.80 ± 0.0	0.80 ± 0.0	ns
FM (kg)	36.2 ± 8.8	37.1 ± 8.5	35.4 ± 9.3	ns
FFM (kg)	56.6 ± 12.6	57.0 ± 12.6	56.3 ± 12.9	ns
MM (kg)	52.9 ± 13.9	52.2 ± 15.7	53.5 ± 12.4	ns
TBW (%)	44.4 ± 5.2	43.9 ± 5.8	45.0 ± 6.4	ns
**24 h Blood Pressure**				
SBP (mmHg)	119 ± 10	118 ± 9	121 ± 12	ns
DBP (mmHg)	73 ± 8	72 ± 8	74 ± 9	ns
MAP (mmHg)	89 ± 9	88 ± 8	91 ± 9	ns
**Laboratory parameters**				
Glucose (mmol/L)	5.4 ± 0.5	5.3 ± 0.5	5.6 ± 0.5	ns
TG (mmol/L)	1.5 ± 1.0	1.5 ± 1.1	1.6 ± 0.9	ns
CHOL tot. (mmol/L)	5.4 ± 1.0	5.4 ± 1.2	5.4 ± 0.9	ns
LDL (mmol/L)	3.2 ± 0.9	3.3 ± 0.9	3.1 ± 0.9	ns
HDL (mmol/L)	1.6 ± 0.4	1.6 ± 0.4	1.6 ± 0.3	ns
ALAT (U/L)	31 ± 23	30 ± 29	31 ± 18	ns
ASPAT (U/L) *	20 (16; 24)	22 (17; 23)	19 (16; 27)	ns
Creatinine (µmol/L)	53.0 ± 8.8	61.9 ± 17.7	53.0 ± 8.8	ns
Uric acid (µmol/L)	309 ± 71	303 ± 48	315 ± 89	ns

Note: BMI—body mass index; WHR—waist/hip ratio; FM—fat mass; FFM—fat-free mass; MM—muscle mass; TBW—total body water; SBP—systolic blood pressure; DBP—diastolic blood pressure; MAP—mean arterial pressure; TG—triglycerides; Chol. tot—total cholesterol; LDL—low-density lipoprotein cholesterol; HDL—high density lipoprotein cholesterol; ALAT—alanine aminotransferase; ASPAT—aspartate aminotransferase; * results presented as the median. Statistical significance: ns—nonsignificant.

**Table 2:** The mean dietary macronutrient composition of the habitual diets at baseline and of the energy-restricted LGI and HP diets (based on the food diaries).

Nutrients Daily Intake	Habitual Diet	LGI Diet	HP Diet	Norms Recommended for the Polish Population [33]
Energy intake (KJ)	7503 ± 2215	5134 ± 768	5087 ± 966	Individual, depending on different factors
Energy intake (Kcal)	1792 ± 528	1225 ± 184	1213 ± 231	
Protein (g)	82 ± 27	74 ± 11	91 ± 13	
Protein (%) Protein (g/kg b.m.)	18 ± 4 0.88 ± 0.3	24 ± 3 0.83 ± 0.3 *	30 ± 4 1.02 ± 0.3 *	10–20% 0.9 g/kg/b.m.
Fat (g)	78 ± 31	42 ± 9	42 ± 14	
Fat %	39 ± 7	31 ± 4	31 ± 4	20–35%
Carbohydrates (g)	192 ± 69	137 ± 29	118 ± 26	
Carbohydrates%	43 ± 10	45± 5	39 ± 3	45–67%
Fatty acids saturated (g)	27 ± 10	16 ± 6	15 ± 6	not specified
Fatty acids monosaturated (g)	32 ± 14	15 ± 4	16 ± 5	
Fatty acids polyunsaturated (g)	12 ± 7	8 ± 2	8 ± 3	
Cholesterol (mg)	369 ± 186	242 ± 97	290 ± 123	300 mg/d

Note: * results are approximated as the diaries were completed during 3 different days of 4 week periods between test weightings; b.m.—body mass.

**Table 3:** The changes in body mass and body composition—during the 8-week period of energy-restricted diets: 4-week high protein (HP) or low glycemic index (LGI) diet given in a random order and followed by the opposite diet.

Variable	HP-LGI Group (*n* = 17)	LGI-HP Group (*n* = 18)	Statistics
Body mass (kg) week 0	94.1 ± 13.6	91.7 ± 17.1	ns
Body mass (kg) week 4	90.2 ± 13.1 ^a^	88.0 ± 16.0 ^a^	ns
Body mass (kg) week 8	88.1 ± 12.7 ^a, b^	86.9 ± 15.6 ^a, b^	ns
			
FM (kg) week 0	37.1 ± 8.5	35.4 ± 9.3	ns
FM (kg) week 4	34.2 ± 7.7 ^a^	33.1 ± 8.9 ^a^	ns
FM (kg) week 8	32.4 ± 7.9 ^a, b^	32.3 ± 9.6	ns
			
FFM (kg) week 0	57.0 ± 12.6	56.3 ± 12.9	ns
FFM (kg) week 4	54.2 ± 15.3	54.8 ± 12.1 ^a^	ns
FFM (kg) week 8	55.7 ± 11.7 ^a^	54.5 ± 12.7 ^a^	ns
			
MM (kg) week 0	52.2 ± 15.7	53.5 ± 12.4	ns
MM (kg) week 4	53.2 ± 12.0	52.0 ± 11.7 ^a^	ns
MM (kg) week 8	53.0 ± 11.3	51.9 ± 12.3 ^a^	ns
			
TBW (%) week 0	43.9 ± 5.8	45.0 ± 4.6	ns
TBW (%) week 4	45.2 ± 5.4 ^a^	45.6 ± 4.9	ns
TBW (%) week 8	46.2 ± 5.2 ^a, b^	46.0 ± 5.6 ^c^	ns

Note: FM—fat mass; FFM—fat-free mass; MM—muscle mass; TBW—total body water. Statistical significance: ^a^ *p* < 0.01 vs. week 0; ^b^ *p* < 0.01 vs. week 4; ^c^ *p* < 0.02 vs. week 0, and ns—nonsignificant.

**Table 4:** The changes in laboratory parameters during the energy-restricted HP and LGI diets.

Variable	HP-LGI Group (*n* = 17)	LGI-HP Group (*n* = 18)	Statistics
Glucose (mmol/L) week 0	5.3 ± 0.5	5.6 ± 0.5	ns
Glucose (mmol/L) week 4	5.2 ± 0.8	5.2 ± 0.4	ns
Glucose (mmol/L) week 8	5.4 ± 0.7	5.4 ± 0.9	ns
			
TG (mmol/L) week 0	1.5 ± 1.1	1.6 ± 0.9	ns
TG * (mmol/L) week 4	0.8 (0.7; 1.2)	1.5 ± 1.3	ns
TG (mmol/L) week 8	1.1 ± 0.3	1.5 ± 0.9	ns
			
CHOL. total (mmol/L) week 0	5.4 ± 1.2	5.4 ± 0.9	ns
CHOL. total. (mmol/L) week 4	4.9 ± 1.3 ^a^	5.0 ± 1.0 ^a^	ns
CHOL. total (mmol/L) week 8	5.0 ± 1.3	5.1 ± 1.1 ^a^	ns
			
LDL (mmol/L) week 0	3.3 ± 0.9	3.1 ± 0.9	ns
LDL (mmol/L) week 4	2.8 ± 0.8 ^a^	2.9 ± 0.3	ns
LDL (mmol/L) week 8	3.0 ± 1.0 ^b^	2.9 ± 0.3	ns
			
HDL (mmol/L) week 0	1.6 ± 0.4	1.6 ± 0.3	ns
HDL (mmol/L) week 4	1.5 ± 0.5	1.5 ± 0.3	ns
HDL (mmol/L) week 8	1.5 ± 0.4	1.6 ± 0.3	ns
			
ALAT (U/L) week 0	30 ± 29	31 ± 18	ns
ALAT (U/L) week 4	29 ± 19	26 ± 13	ns
ALAT * (U/L) week 8	19 (16; 29)	27 ± 15	ns
			
ASPAT * (U/L) week 0	22 (17; 23)	19 (16; 27)	ns
ASPAT * (U/L) week 4	23 (20; 26)	20 ± 5	ns
ASPAT * (U/L) week 8	20 (16; 25)	20 ± 5	ns
			
Creatinine (µmol/L) week 0	61.9 ± 17.7	53.0 ± 8.8	ns
Creatinine (µmol/L) week 4	61.9 ± 17.7	53.0 ± 8.8	ns
Creatinine (µmol/L) week 8	61.9 ± 3.5	53.0 ± 3.5	ns
			
Uric acid (µmol/L) week 0	303 ± 48	315 ± 89	ns
Uric acid * (µmol/L) week 4	291 (243; 321)	303 (262; 381) ^a’^	ns
Uric acid * (µmol/L) week 8	303 ± 54	268 ± 83 ^b’^	ns

**Note:** TG—triglycerides; Chol. tot—total cholesterol; LDL—low-density lipoprotein cholesterol; HDL—high density lipoprotein cholesterol; ALAT—alanine aminotransferase; ASPAT—aspartate aminotransferase; * results presented as the median. Statistical significance: ^a^ *p* < 0.01 vs. week 0, ^b^ *p* < 0.04 vs. week 4, ^a’^ *p* < 0.02 vs. week 0, ^b’^ *p* < 0.01 vs. week 4, ns—nonsignificant.

## Data Availability

The data presented in this study will be made available from the authors upon reasonable request.
